# Global and Local Visual Processing in Rate/Accuracy Subtypes of Dyslexia

**DOI:** 10.3389/fpsyg.2020.00828

**Published:** 2020-04-30

**Authors:** Yael Goldstein-Marcusohn, Liat Goldfarb, Michal Shany

**Affiliations:** Department of Learning Disabilities, Edmond J. Safra Brain Research Center for the Study of Learning Disabilities, University of Haifa, Haifa, Israel

**Keywords:** dyslexia, global and local processing, visual span, reading, learning disabilities

## Abstract

Words are processed in both a global and local manner. Studies on global versus local processing styles in individuals with and without dyslexia are inconclusive. In the present study, we investigated whether distinct patterns of global/local visual processing were associated with more precisely defined dyslexia profiles. Previous studies on dyslexia provide evidence of accuracy- and rate-based subtypes, with impairment in one dimension alongside normal performance in the other. In the current study, three groups of adult readers: rate disability, accuracy disability, typical development, were presented with nonlinguistic global /local congruency task. The results revealed that the rate disability group had deficiencies performing the global task while the accuracy disability group had deficiencies in the local task. These results are discussed in the context of global/local word processing and in relation to dyslexia. Specifically, they suggest that different patterns of global/local processing are observed between different types of dyslexics, and imply that practitioners should modify their treatment based on the specific deficiency.

## Introduction

### Subtypes of Dyslexia

Dyslexia is a specific learning disability, neurobiological in origin, characterized by difficulties with accurate and/or fluent word recognition and by poor spelling and decoding (Lyon et al., 2003).

Past hypotheses have opined that Dyslexia is underlined by a phonological deficit (Shankweiler and Liberman, 1989). However, these hypotheses failed to explain the full range of reading disability profiles (Share and Stanovich, 1995). In recent years, it has become clear that at least some aspects of phonological processing (such as phonological awareness) may be relatively unimpaired in some readers with disabilities (Wolf and Bowers, 1999; O’Brien et al., 2012), that is even evident in differences in neural processing of tones and phonemes (Lachmann et al., 2005), raising the possibility that multiple sources of reading disability exist (Ramus et al., 2003; Pennington, 2006). This notion of heterogeneity among individuals with dyslexia has set off various attempts to delineate subtypes of reading disability (e.g., Boder, 1970; Mattis et al., 1975; Lyon et al., 1982; Stanovich and Siegel, 1994; Fletcher et al., 1997; Morris et al., 1998).

A most influential subtyping framework derives from Coltheart et al.’s (1993) dual-route model of word reading, which allows for descriptions of dyslexia deficits localized at the lexical, and/or nonlexical levels. The dual-route model suggests that skilled readers have two separate cognitive reading procedures at their disposal: the lexical route, which is a dictionary lookup procedure, and the nonlexical or sublexical route, which is a letter-to-sound conversion rule procedure. Subsequently, dyslexia is the outcome of a deficit in one or more of these routes, as different deficits result in different types of reading disabilities. According to this model, there are at least three subtypes of dyslexia: surface, phonological and combined surface-phonological, respectively (Coltheart et al., 2001).

Another subtyping scheme that has received considerable attention is Wolf and Bowers’ double-deficit hypothesis (1999) which proposes that phonological awareness (PA) and rapid-naming (RAN) deficits are separable and distinct reading impairments. Accordingly, three subtypes of dyslexics are posited: an exclusively PA impaired group, an exclusively RAN-deficit group, and a third group with impairments in both skills. Many studies support the double-deficit hypothesis of dyslexia, which posits that rapid naming is an additional coredeficitthat can cause reading difficulties, with or in the absence of phonological impairments and that fluency differentiates between disabled and intact readers (Wimmer, 1993; Yap and Van der Leij, 1993; Breznitz, 1997; Cossu, 1999a, b; Zoccolotti et al., 1999; de Jong and van der Leij, 2003; Lyytinen et al., 2004; Leppanen et al., 2006; Nikolopoulos et al., 2006).

### Rate/Accuracy Subtyping Frameworks

In recent years, there has been a growing number of studies suggesting that dyslexics may be selectively impaired only in reading accuracy or only in reading rate (Lovett, 1984, 1987; Leinonen et al., 2001; Shany and Share, 2010; Shany and Breznitz, 2011). Evidence of this subtyping scheme has even been shown in differences in gray matter patterns between dyslexics with deficits in rapid naming compared to those with deficits in phonological processing (Jednoróg et al., 2014). Lovett (1984, 1987) was the first to classify a clinical sample of dyslexics (ages 8–13) into accuracy-disabled and rate disabled subtypes. She compared accuracy-impaired and rate-impaired children in various linguistic tasks. However, her findings showed that the accuracy-impaired group was not purely accuracy-disabled and also showed impairment in reading rate. Lovett proposed that the two subgroups represent different points on a developmental continuum of reading acquisition. Another study was conducted by Leinonen et al. (2001) on adult readers of a shallow orthography (Finnish), identified a rate-only disabled (“hasty”) subgroup in a sample of 84 Finnish adult dyslexics, in addition to an accuracy-only (“hesitant”) disabled subgroup. A third subgroup was severely impaired on both rate and accuracy. However, true double dissociation between the groups was not confirmed, because both subgroups displayed impairment of differing degrees on both rate and accuracy, which raises the argument that some hold, that reading disabled readers vary across a spectrum rather than hold distinct reading profiles.

The only studies who were able to show a distinct double dissociation between reading rate and accuracy, which remained across various reading tasks, were conducted on the Hebrew orthography (Shany and Share, 2010; Shany and Breznitz, 2011; Elkayam, 2016). Shany and Breznitz (2011) reported evidence for a true double dissociation between accuracy and rate in a nationally representative sample of Hebrew-speaking fourth graders. Two “hard” single-deficit groups were identified with selective deficits in either rate or accuracy in the presence of intact performance on the non-impaired dimension. Furthermore, the two subgroups also differed in their cognitive–linguistic profiles; the accuracy-only subgroup was poor on phonological awareness as well as a number of linguistic measures (but not RAN), whereas the rate-disabled subgroup was impaired only on RAN. Those results were replicated in a national representative sample of grades 2 and 6 (Elkayam, 2016). In a follow-up study, Shany and Breznitz (2011) examined 345 students with dyslexia. A selective reading accuracy deficit was found to be associated with language and cognitive abilities affecting verbal memory, phonological awareness, morphological and orthographic knowledge, but not rapid automatized naming. A selective reading rate deficit was found to be associated with RAN, rapid automatized naming of print related material (visual-linguistic task), but not in the fidelity of their verbal/linguistic knowledge.

### Visual Attention and Dyslexia

Since the 1990s, evidence has been accumulating that individuals with dyslexia have various deficits in visual attention (VA). These deficits include difficulties in: performing serial search tasks (Williams et al., 1987; Casco and Prunetti, 1996; Vidyasagar and Pammer, 1999; Iles et al., 2000), selective attention (Richards et al., 1990; Casco et al., 1998), orienting attention using a peripheral cue (Brannan and Williams, 1987), suppressing information from the periphery of the visual field (Geiger and Lettvin, 1987; Geiger et al., 1994), and sustaining attentional focusing (Facoetti et al., 2000) among others. Additionally, some studies even suggest that a VA span deficit might contribute to developmental dyslexia, independently of a phonological disorder (Bosse et al., 2007; Vidyasagar and Pammer, 2010). On the other hand, some suggests that under some conditions individuals with dyslexia can outperform individuals with typical development. For example, Schneps et al. (2012) found that individuals with dyslexia perform better in a low-pass filtered natural scenes task in which the perception for low spatial frequency components is examined.

While VA was included in the range of cognitive areas addressed in the study of RD subtypes, few in-depth attentional processes were examined. Shany and Breznitz (2011) examined the performance of rate-disabled, accuracy-disabled, and double-deficit subgroups using the d2 Test of Attention (Brickenkamp, 1962). In this task, participants are asked to cross out all instances of a specific target character, which are interspersed among non-target characters, in 14 successive timed trials (Brickenkamp, 1962). The accuracy-disabled subgroup performed this task significantly worse than did the rate-disabled group, the double deficit group, and skilled readers. Shany and Breznitz also examined inhibition abilities in each of these groups using a version of the Stroop Color and Word Task (Golden, 1978). The task had three parts: a word page, in which color names were printed in black ink; a color page, in which semantically meaningless symbols were printed in colored ink; and a color-word page, which was comprised of the words from the first page, printed in the colors from the second page, with the restriction that word and color do not match. Participants were instructed to move down the columns, reading words or naming ink colors as quickly and accurately as possible, within a given time limit. The Stroop effect (or interference effect), calculated as the reaction time ratio between word naming and color-word naming, was found to be larger in the accuracy-deficit and double-deficit subgroups than in the rate deficit and skilled reader subgroups, indicating a lesser ability to inhibit task-irrelevant information.

The current study aimed to extend the investigation of attentional problems that the two RD subgroups may have and examine a potential disassociation between the groups by examining their global and local attentional abilities.

### Global/Local Visual Processing

It has been suggested that people can act in two different attention modes- global and local (e.g., Förster et al., 2008; Förster and Dannenberg, 2010). When the global system is activated, people perceive gestalts, activate broad categories in memory, and integrate incoming information into existing knowledge structures, whereas when the local attention system is activated, people perceive details, and activate narrow categories that typically lead to exclusion of incoming stimuli. Global and local processing is usually manipulated using a Navon or Navon -like tasks. Navon (1977), originally used hierarchical stimuli consisting of a *global* letter composed of either congruent or incongruent *local* letters (e.g., a larger *H* made up of smaller *H*s or *Y*s, respectively). Participants had to identify either the larger character (*global task*) or the smaller ones (*local task*). In the local task, an incongruent global letter has repeatedly been shown to inhibit responses relative to a global congruent letter. Meanwhile, in the global task, the results are less robust and only some experiments have shown interference stemming from an incongruent local letter as compared to a congruent local letter (Tyler and Tucker, 1982; Mottron et al., 2003; Yovel et al., 2005).

The results of the Navon experiment have been replicated numerous times over the years using different variations, including stimuli that are not linguistic. For example, in one variation, Weinbach and Henik (2011) showed participants a large arrow made up of small arrows, which pointed in either the same (congruent) or different (incongruent) directions. Participants had to indicate whether the large arrow (global task) or the small arrows (local task) pointed left or right. The findings revealed that in both the local and the global tasks, reaction times in incongruent trials were slower than reaction times in congruent trials and that participants experienced greater interference by global information in the local task than vice versa. As the magnitude of the interference in each task is taken to reflect the automatic activation of the interfering dimension (e.g., Tzelgov, 1997), these findings demonstrate greater automatic activation of global information.

Global/local perception can differ between different populations. For example, compared to healthy participants, participants with autism (Mottron et al., 2003), obsessive-compulsive personality (Yovel et al., 2005), and anxiety (Tyler and Tucker, 1982) have been shown to exhibit enhanced local processing. Moreover, experiences can alter global or local perception. Global processing, for example, is more likely to occur when people are exposed to unfamiliar as opposed to familiar events (Förster and Dannenberg, 2010).

### Global/Local Processing in Reading

The concepts underlying the global/local distinction can also be applied to reading, as words are comprised of both global and local components. For example, one study supporting local processing in word recognition showed that the letters of a word must be sufficiently spaced so that each letter can be processed separately in order for the word to be recognized (Martelli et al., 2005). On the other hand, there is evidence that fast expert readers exhibit signs of holistic word reading, such as identifying clusters of letters based on regularities found in words (Orbán et al., 2008). Furthermore, letters are better recognized in the context of a word than in isolation, in an effect commonly known as the *word superiority effect* (Reicher, 1969).

Cohen et al. (2008) showed that word reading can rely both on part-based and holistic processing, depending on the context. According to this conception, expert holistic processing of words is subserved by a ventral occipitotemporal pathway, with more anterior regions (visual word form area) responsible for holistic processing (parallel encoding). Part-based processing, meanwhile, is subserved by the dorsal pathway and enables serial attention to letters when the context is not optimal for whole word reading. Children who have not developed a sufficient level of expertise in reading are thought to rely more heavily on the dorsal route. Meanwhile, it is believed that expert readers display a more holistic word reading style. It has also been suggested that the local letter by letter processing could handle reading all types of words accurately. However, the global whole word reading process is faster. Hence when global processing can achieve a sufficient accuracy level this process can aid the local one (e.g., Pelli and Tillman, 2007).

Another framework addressing global-local processing in context of reading is the *functional coordination* approach (Lachmann and van Leeuwen, 2014). According to this theory, reading recruits the analytic (local) strategy of visual processing, which existed prior to literacy but became the preferred method of letter processing. In their study, Lachmann et al. (2014) examined global/local processing of letters compared to non-letters using the Navon task, when the hierarchical figure is presented with dimensions similar to those of written text. Under these conditions, they have found that the global precedence effect appears only for non-letters but not for letters. According to their interpretation, letters are processed using an analytic strategy, while non-letters recruit global processing, as hierarchical stimuli.

Given the global and local components of word reading, there is clear value in comparing global and local processing among readers with and without RD. However, few studies attempted to do so. Matthews and Martin (2009) examined differences between good and poor phonological decoders in English with respect to the allocation of spatial attention to global and local levels of hierarchical stimuli during a sustained attention task. The results suggest that poor decoders showed longer reaction times than did good decoders in both tasks, and tended to be less accurate in the global task. Similar results were reported in a more recent study by Franceschini et al. (2017). In a series of five experiments in 353 primary school children, utilizing the Navon task to evaluate global and local processing, researchers have found: (i) children with dyslexia showed no interference from the global information in the local task and presented a larger interference of the local incongruent feature in the global perception task, compared to control; (ii) global before local perception trainings improve reading skills in children with dyslexia; and (iii) pre-reading local before global perception longitudinally predicts future poor readers. However, another study (Von Karolyi, 2001) examining English readers indicated that on other global/local-like tasks, individuals with RD were more inclined toward global processing and more deficient in local processing when they were compared to intact readers. Specifically, participants with RD were compared to controls on two computer-based visual-spatial tasks. The first was an impossible figures task designed to assess global processing. In this task, participants were asked to determine whether a figure was possible or impossible, which could only be achieved by assessing it holistically. The second task was the Celtic Matching Task, designed to assess local processing. Participants were asked to select from a set of figures the figure that was identical to a target figure, requiring attention to small details. The study’s findings revealed that the RD group was faster than the control group at identifying impossible objects in the global processing task, while controls outperformed the RD group on the detail-oriented local processing task.

To summarize, findings on global versus local processing styles in individuals with and without RD are thus far inconclusive. It should be noted, however, that past research has not differentiated between different RD subtypes, which may shed light on discrepancies between the results of different studies. As previous work has shown differences between RD subgroups with respect to numerous cognitive functions (Shany and Share, 2010; Shany and Breznitz, 2011), attention included, it is possible that the subgroups differ in global and local processing as well, and that testing them as one made it difficult to observe patterns of interference specific to each subgroup.

### Goals and Hypotheses

The current study examined three groups of adult Hebrew readers: adults with a selective reading rate deficit (rate disability subgroup), adults with a selective deficit in reading accuracy (accuracy disability subgroup), and intact readers. The objective was to examine global and local processing among these reader groups using non-verbal hierarchical stimuli. As in Weinbach and Henik (2011), participants were presented with a large arrow comprised of smaller arrows, under both congruent and incongruent conditions, and asked to perform global (large arrow) and local (smaller arrows) processing tasks. As noted above, words are comprised of both global and local components and deficiencies in the different mechanisms can lead to a different profile of reading impairment. Deficiency in the local detailed processing could lead to more reading errors and deficiency in the general global processing could lead to speed reading impairment. Hence we hypothesize that if indeed the general global -local mechanism of attention is related to reading and dyslexia than readers with rate disabilities would be more deficient in the global processing and readers with accuracy disabilities would be more deficient in the local processing.

## Materials and Methods

### Participants

A total of sixty university students participated in the experiment, twenty in each of the three experimental groups: (a) control group of intact readers (mean age = 29.75, SD = 3.14; 45% female); (b) rate disability RD group (mean age = 29.35, SD = 3.51; 70% female); and (c) accuracy disability RD group (mean age = 29.15, SD = 4.2; 60% female). All group members reported having no prior diagnosis of dyscalculia or attention-deficit/hyperactivity disorder (ADHD). The three groups did not differ significantly with respect to age, *F*(2,57) = 0.142, *p* = 0.869, ns, or gender, *X*^2^(2,1) = 2.6, *p* = 0.27, ns. All participants were native Hebrew-speakers with normal or corrected-to-normal vision.

Participants in the RD groups all had diagnoses of RD given by a learning disability expert and had undergone a computerized battery of standard tests and questionnaires (MATAL Battery, 2007) at the University of Haifa Learning Disability Assessment Unit between the years 2008 and 2013. They were recruited from lists available at the Assessment Unit. Only participants who had signed a consent form allowing researchers to screen their assessment files and contact them were contacted by phone and asked to participate in the current study. The control group was recruited via ads placed in a number of institutions of higher learning throughout Israel. All participants signed consent forms and received 50 NIS for their participation.

### Grouping Measures

Participants with reading disabilities were divided between the two subtype groups based on the rate and accuracy measures of two tasks, as detailed below. To be included, participants had to meet the criteria defined for both tasks.

#### Vocalized Pseudoword Reading Task

As noted above, all students in the RD groups had previously taken the MATAL Battery (2007), which includes a pseudoword reading task comprised of 25 nonwords, each consisting of two to three syllables. The words are representative of the rules of the Hebrew language and include a variety of morphological patterns, consonants, and vowels. In the task, participants were instructed to read the words as quickly and accurately as possible. Scoring was based on the number of words read accurately (words per minute) and on reading rate (percentage of correctly pronounced words). In an initial sample compiled by the Israeli National Institute for Testing and Evaluation, Cronbach’s alpha was 0.945 for accuracy and 0.719 for rate. To be included in the current study, participants with RD had to show a dissociation between rate and accuracy scores. Specifically, participants were included in the rate disability subgroup if they scored below the 16th percentile in rate and above the 50th percentile in accuracy (mean rate percentile = 13.4, mean accuracy percentile = 64.6), and in the accuracy disability subgroup if they scored below the 16th percentile in accuracy and above the 50th percentile in rate (mean rate percentile = 69.8, mean accuracy percentile = 11.5^1^). Note that in Israel, both in research and clinical work, it is custom to use one standard deviation below the average reading score in order to diagnose reading difficulties.

#### Isolated Unpointed Word Reading Task

All participants with RD underwent an additional task involving vocalized reading of a list of 30 isolated unpointed (not fully vowelized) nouns representing different frequency levels, lengths, and morphological structures. The measure is considered standard for identifying dissociations between rate and accuracy in individuals with RD in shallow orthographies (e.g., Wimmer, 1993; Breznitz, 1997; Cossu, 1999a, b; Zoccolotti et al., 1999; de Jong and van der Leij, 2003; Lyytinen et al., 2004; Leppanen et al., 2006; Nikolopoulos et al., 2006). Participants were instructed to read the words as quickly and accurately as possible. Scoring was based on the number of words read accurately and on reading rate. In an initial sample compiled by the National Institute for Testing and Evaluation, Cronbach’s alpha was 0.75. Readers were included in the rate disability group if their standardized score was under -1.5 in rate and over -0.25 in accuracy (mean rate = 31.34 s, mean standardized score for rate = -2.3, mean number of correct words = 28.85, mean standardized score for accuracy = 0.2), and in the accuracy disability group if they scored under -1.5 in accuracy and over -0.25 in rate (mean rate = 20.25 s, mean standardized score for rate = -0.1, mean number of correct words = 24.83, mean standardized score for accuracy = -2.5).

The control group also performed the isolated word reading task to verify intact reading in terms of both rate and accuracy (mean rate = 22.22 s, mean standardized score for rate = 0.4, mean number of correct words = 29.03, mean standardized score for accuracy = 0.1).

### Global/Local Processing Measure

The experiment was run on a Lenovo Yoga 2 computer with a 13-inch screen. E-Prime software version 2.0 was used for programming, presentation of stimuli, and timing operations. Responses were collected through the computer keyboard.

The task employed in the current study was similar to the global/local task introduced by Weinbach and Henik (2011), described generally above. Visual stimuli were black figures presented in the center of a screen on a white background. The fixation stimulus was a plus sign and subtended a 0.5° visual angle. The global figures were arrows made up of smaller arrows pointing to the left or right and subtended a visual angle of 8.5°. The local figures were the smaller arrows, which were spatially organized to create the global figure, and subtended a 1° visual angle. This setup created two congruency levels: congruent—when the large arrow and the smaller arrows pointed in the same direction, and incongruent—when the large arrow and the smaller arrows were pointed in opposite directions. Half of the trials were congruent and half were incongruent.

### Procedure

Participants were seated 60 cm from a computer screen and asked to indicate the direction of the small arrows (local task) and ignore the large arrow or to indicate the direction of the large arrow (global task) and ignore the smaller ones. The order of the two tasks was counterbalanced, such that half of the participants performed the global task first and the local task second, and the other half performed the tasks in the opposite order. The time frame was the same for both the global and local tasks. Each trial began with a fixation cross (“+”) presented in the center of the screen for 500 ms, which was then replaced by the arrow target stimulus. The target remained in view until the participant responded or until 3,000 ms had passed. After a response was made, the stimulus disappeared and after 500 ms the next step began. Each task (global and local) started with a training block of 10 trials that were selected randomly from the full set of trials, followed by 80 experimental trials.

## Results

The mean RT for correct responses, for each participant in each condition, was calculated. Trials post erroneous responses and over 2.5 standard deviation of the mean reaction time (RT), were excluded from the analysis (4.7% equally distributed across conditions). Mean accuracy and RT were not correlated, *r*(58) = 0.211, *n* = 60, *p* = 0.105, and error rates were relatively low (4%), therefore only RT analysis was performed, as it creates a statistical floor effect so the error analysis is not expected to be significant.

### Global Task: Reaction Time Analysis

**FIGURE 1:**
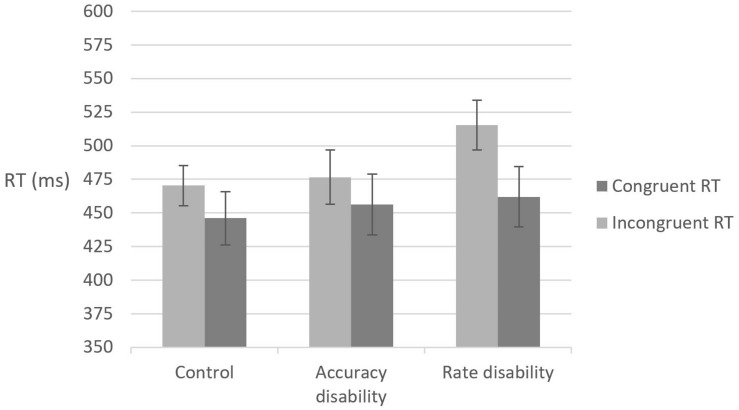
Reaction time (RT) means and standard error for the global task in each study group under the different congruency conditions.

The mean reaction time for correct responses for each participant in each condition was calculated (see Figure 1) and two-way analysis of variance was conducted, with congruency as the within-subject variable and group as the between-subject variable. A main effect of congruency was found, *F*(1,57) = 60.9, MSE = 21,015, *p* < 0.01, but not for group, *F* < 1. Importantly, a significant interaction between group and congruency was found, *F*(2,57) = 4.06, MSE = 1,397, *p* < 0.05. Further analysis revealed that the magnitude of the congruency effect (incongruent-congruent) was larger in the rate disability group than in the accuracy disability group, *t*(38)=2.3, *p* < 0.05 and the control group, *t*(38) = 2.45, *p* < 0.05. The accuracy disability group did not differ significantly from the control group in the magnitude of the congruency effect, *p* = 0.85. These results indicate that the rate disability group, as compared to the other two study groups, had greater difficulty ignoring automatic local interference in the global task.

### Local Task: Reaction Time Analysis

**FIGURE 2:**
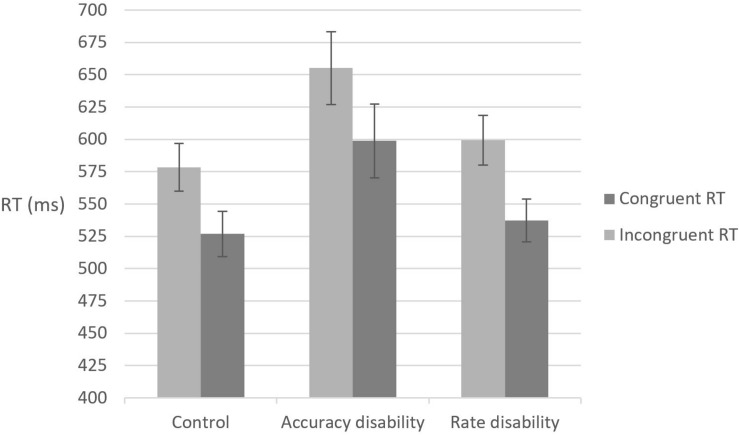
Reaction time (RT) means and standard error for the local task in each group under the different congruency conditions.

The mean reaction time for correct responses for each participant in each condition was calculated (see Figure 2) and a two-way analysis of variance was conducted, with congruency as the within subject variable and group as the between subject variable. A main effect of congruency was found, *F*(1,57) = 102.6, MSE = 64,955, *p* < 0.001, a marginally significant effect was found for group, *F*(2,57) = 2.64, MSE = 37,202, *p* = 0.051, and no significant interaction effect was found, *F* < 1. Further analysis for the main effect of group indicated that the accuracy disability group was significantly slower than the control group, *t*(38)=2.005, *p* < 0.05, and was significantly different from the rate disability group, *t*(38) = 1.76, *p* < 0.05. There was no significant difference between the rate-disabled group and the control group, *t*(38) = 0.46, *p* = 0.65. These results indicate that the accuracy disability group, as compared to the other two study groups, had greater general difficulty performing the local task.

Lastly, the relationship between the global/local task was examined. Hence a three-way analysis of variance was conducted, with congruency and task as within subject variables and group as the between subject variable. Main effects were found for task, *F*(1,57)=109.04, MSE = 611,922, *p* < 0.01, with overall faster response times for the global compared to the local task, and congruency, *F*(1,57) = 164.75, MSE = 79,932, *p* < 0.01, as response times for congruent targets were faster compared to incongruent targets. Additionally, a significant interaction between task and congruency revealed that the congruency effect was larger in the local task than the global task, *F*(1,57) = 12.26, MSE = 6,038, *p* < 0.05. Finally, a significant interaction between task and group, *F*(1,57) = 3.72, MSE = 20,852, *p* < 0.05, and a marginally significant triple interaction between task, congruency and group, *F*(2,57) = 3.04, MSE = 1,498, *p* = 0.056, indicate differences in response time patters in the different condition between groups.

Further analysis suggests that the difference in RT between the global task and the local task was larger for the accuracy disability group than the rate disability group [*F*(1,57) = 6.57, MSE = 5612, *p* < 0.05) and the control group [*F*(1,57) = 4.33, MSE = 5,612, *p* < 0.05). This was due to the slowness in the local task in the accuracy disability group as was described in the analysis above. The difference in RT between the global task and the local task was the same between the rate disability group and the control group *F* < 1.

## Discussion

The current study was designed to assess the global and local processing styles of individuals with rate-specific and accuracy-specific subtypes of dyslexia. This was tested using traditional hierarchical stimuli, in which participants had to identify the global figure in the global task and the local figure in the local task. We hypothesized that the slowness of the rate disability subtype would be associated with a more part-based (local) reading style and with less use of global processing. Conversely, we hypothesized that in the fast and inaccurate profile of the accuracy disability subtype would be associated with less local processing. The results revealed the assumed disassociation: the rate disability group had more difficulty with the global task and the accuracy disability group, had more difficulty with the local task. Also note that the pattern of difficulties within the tasks differed between the groups. The rate disability group in the global task had more difficulty to ignore the local processing, as they experienced a greater congruency effect that was driven by the extra attention given to non-relevant local elements (i.e., difficulties to ignore the local dimension). In the reading domain, this can be connected to problems concerning the extra attention given to local elements when the global and fast reading can be performed instead. This extreme processing of local elements of the words does not cause any error in reading however, it makes the reading processes slower. As noted by Pelli and Tillman (2007), the local reading processor could read all types of words accurately but the global processor is faster hence it is more beneficial to switch to this style when it can be applied. A more part-based manner of reading involving disassembly of words into their components rather than recognition of words as complete orthographic patterns could lead to slow but accurate reading. Regarding the accuracy disability group, note that they showed significantly slower local task performance beyond the congruent and incongruent conditions as compared to the other two groups. In addition this slowness was also reflected in the difference in RT between the global task and the local task. This difference was the largest for the accuracy disability group. Slower local task performance indicating difficulties in general local processing. The general deficiency of attending to local elements might cause those individuals not to attend to local elements of the words such as word segments, roots and affixes which in turn lead to reading errors but not to a speed deficiency. It is possible that this group shifted to fast reading (a more holistic global reading style) before the development of proper expertise in the primary, part-based (local) reading manner, leading them to read quickly, like intact readers, but not accurately.

Past research on global/local processing in RD has not yielded conclusive results. Some studies have shown that individuals with RD are inclined toward global processing, as are intact readers (Keen and Lovegrove, 2000), while others suggest that they are more inclined toward local processing (Von Karolyi, 2001; Franceschini et al., 2017). However, none of these studies has differentiated between RD subtypes.

The RD group examined by Matthews and Martin (2009) was chosen on the basis of decreased accuracy measures on a non-word reading test, and can hence be considered accuracy-disabled. In that study, the RD group tended to be less accurate on the global task than were skilled readers, which can seem inconsistent with the current findings, which indicated that readers in the accuracy disability group were relatively deficient in local processing. However, as the Matthews and Martin study did not specifically exclude readers who also had deficits in reading rate, it is likely that their sample included participants with deficits in both accuracy and rate.

Our results might support the findings reported by Shany and Breznitz (2011) with respect to the d2 Test of Attention (Brickenkamp, 1962), on which the accuracy disability group preformed significantly worse than the rate disability group, which did not differ from the skilled reader group. A similar task to the D2 is the connect-the-dots task, in which participants are given 1 min to complete a picture by connecting numbered dots in ascending order. Both tasks require great attention to detail so it is possible that they also required local attention. Interestingly, Friedman et al. (2003) found that local priming facilitates more accurate completion of a connect-the-dots task, while global priming impairs performance. Since accurate completion of the d2 task might also require narrowed perceptual attention, it is likely to be linked to local processing tendencies. Thus, difficulties in global processing in the rate disability group, as revealed in the current study, might explain the impaired performance reported on the d2 task in this RD subtype.

In addition, the current findings also support and strengthen previous research that has proposed a distinction between RD subtypes based on rate and accuracy measures (Lovett, 1984, 1987; Leinonen et al., 2001; Shany and Share, 2010; Shany and Breznitz, 2011). The current findings add to this literature by identifying additional cognitive functions that differentiate between the subtypes, and highlight the possibility that these subgroups can be differentiated not only by their distinctive linguistic profiles, but also by their cognitive profiles.

It is important to note that this study examined adults with RD, making it difficult to gage whether the relevant attentional processes are in fact underlying components of the disability, its byproducts, or a co-occurring phenomenon. Furthermore, as this study examined a Hebrew-speaking population, further research should investigate whether these patterns are apparent in other languages and types of orthography as well. Thus, further research is required to shed more light on the reported phenomenon.

Despite these limitations, the current study has significant implications. First, it opens the door to additional research on attentional mechanisms in RD, and emphasizes the importance of differentiating between attentional processes that characterize RD in general and those that may be restricted to certain subtypes. Second, our findings might help educators optimize intervention methods for students with RD, as there is evidence that tailoring intervention for the type of RD provides better results (Shany, 2014). For example, it may be possible to help readers with accuracy-specific deficits, who struggle in processing local elements, to devote greater attention to word segments, such as roots, affixes, and letter combinations. On the other hand, it may be possible to help readers with rate-specific impairment (who are deficient in processing global elements) by practicing global reading while ignoring local aspects. Finally, experts may use the information provided by the study to generate new assessment measures, to thoroughly understand the nature and extent of learning disabilities among individual readers.

## Data Availability Statement

The datasets generated for this study are available on request to the corresponding author.

## Ethics Statement

The studies involving human participants were reviewed and approved by the University of Haifa ethics committee. The patients/participants provided their written informed consent to participate in this study.

## Author Contributions

LG and MS conceived and designed the study, and edited the manuscript. YG-M collected the data, and wrote the manuscript. Both LG and YG-M analyzed the data.

## Conflict of Interest

The authors declare that the research was conducted in the absence of any commercial or financial relationships that could be construed as a potential conflict of interest.

## Footnotes

1 Note that performance on this subtest of the MATAL Battery is reported in percentiles. Raw scores for the test are converted using a confidential method and not made available in the computerized report.
